# Optimisation and validation of hydrogel-based brain tissue clearing shows uniform expansion across anatomical regions and spatial scales

**DOI:** 10.1038/s41598-019-48460-2

**Published:** 2019-08-19

**Authors:** Adam L. Tyson, Ayesha M. Akhtar, Laura C. Andreae

**Affiliations:** 1 Centre for Developmental Neurobiology, Institute of Psychiatry, Psychology and Neuroscience, King’s College London, London, UK; 2 MRC Centre for Neurodevelopmental Disorders, King’s College London, London, UK; 3 Sainsbury Wellcome Centre for Neural Circuits and Behaviour, University College London, London, UK

**Keywords:** 3-D reconstruction, Fluorescence imaging

## Abstract

Imaging of fixed tissue is routine in experimental neuroscience, but is limited by the depth of tissue that can be imaged using conventional methods. Optical clearing of brain tissue using hydrogel-based methods (e.g. CLARITY) allows imaging of large volumes of tissue and is rapidly becoming commonplace in the field. However, these methods suffer from a lack of standardized protocols and validation of the effect they have upon tissue morphology. We present a simple and reliable protocol for tissue clearing along with a quantitative assessment of the effect of tissue clearing upon morphology. Tissue clearing caused tissue swelling (compared to conventional methods), but this swelling was shown to be similar across spatial scales and the variation was within limits acceptable to the field. The results of many studies rely upon an assumption of uniformity in tissue swelling, and by demonstrating this quantitatively, research using these methods can be interpreted more reliably.

## Introduction

Fluorescence microscopy of fixed tissue sections is widely used in neuroscience, and biomedical science generally. However, light absorption (due to pigmentation) and scatter (due to heterogeneous refractive index (RI) of the tissue) limit the depth of tissue that can be imaged. To overcome this, tissue is usually sliced into thin sections (100 μm or less) which is laborious, and can introduce artefacts if large volumes of tissue are studied.

Light scatter due to lipid content is the predominant mechanism preventing deep imaging in brain tissue, and so tissue-processing methods have been developed to homogenise the RI of the tissue and reduce scatter. These methods are collectively known as tissue clearing, and were originally proposed a century ago^1^. More recently, the idea of tissue clearing for large-volume microscopy has been revisited. These methods have used different approaches, such as immersion in RI matching solutions^2–8^, the use of organic solvents^9–15^ and the direct removal of tissue lipids^16–20^. Of these, the methods relying on lipid removal, and particularly hydrogel-based methods (e.g. CLARITY^17^) have been those most adopted by the research community.

Hydrogel-based tissue clearing methods have so far been popular due to their reliability and flexibility (as they are one of the clearing methods compatible with antibody staining). Many variations on these methods have been published^17,21–27^ but they all share a general core concept. Firstly, the tissue is incubated in a fixative solution containing paraformaldehyde (PFA) and acrylamide (with or without bis-acrylamide). This fixative binds biomolecules containing an amine group (chiefly proteins and nucleic acids) but not membrane phospholipids, and is then polymerised to to form a transparent hydrogel ‘matrix’ within the tissue. As the majority of lipids are not bound to this matrix, they can then be removed by using a detergent solution of sodium dodecyl sulfate (SDS) along with a combination of heat and electrophoresis or mechanical agitation to accelerate the process. Once the sample’s RI is matched using a high RI solution, the final result is a transparent and macromolecule permeable sample in which most protein and nucleic acid is preserved^17,21,27–29^.

There have been tremendous advances in tissue clearing along with imaging and analysis of large volumes of brain tissue. However, because these methods are not as mature as traditional methods (e.g. thin-section immunohistochemistry), two issues remain. The first is choosing an experimental protocol — there are many parameters to choose to ensure effective tissue clearing and staining. The second, and most important, is validation — these methods are starting to become routine, and yet there is very little information about how these methods affect tissue morphology.

Here we present an optimisation of a hydrogel-based tissue clearing and antibody staining protocol in adult mouse brain tissue. This was chosen as it is the most common, and most flexible use of tissue clearing in neuroscience. In addition, a detailed analysis was performed, comparing tissue morphology in cleared tissue to tissue processed using a more conventional method.

## Results

### Tissue clearing

**Figure 1:**
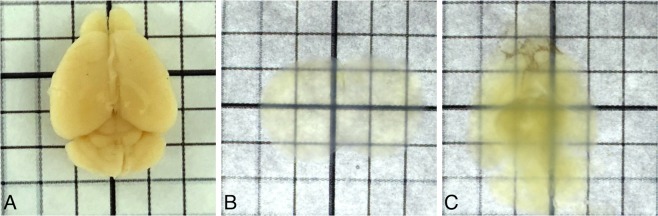
Mouse brain tissue incubated in hydrogel (A4B5P4), cleared using SDS and RI matched using 85% glycerol. (**A**) Mouse brain prior to tissue clearing, (**B**) 2 mm section showing the end point of clearing, (**C**) Cleared whole brain.

To fully optimise hydrogel-based clearing of brain tissue, a number of parameters from the original report^17^ were varied. Samples were incubated whole, in hemispheres, or in slices taken using a brain slicing matrix^30^ and at room temperature or 37 °C with or without shaking in clearing buffer (4% or 8% SDS) to clear. Clearing buffers were changed weekly, until the sample appeared visibly clear (i.e. until the tissue does not obscure printed structures underneath, Fig. 1B).

All tissue samples, regardless of sample size cleared successfully, showing little light scatter despite some discolouration (Fig. 1B). Large volumes of brain tissue (e.g. whole, adult mouse brains, Fig. 1C) could be cleared, despite more discolouration and scatter. A number of hydrogel compositions were tested (Table 1). The specific combinations were chosen based on those used successfully in the literature^17,21,22^, and acrylamide/PFA concentration was varied together to avoid the need to test the large number of combinatorial options. We found that shaking at 37 °C was required for complete, uniform clearance, but that hydrogel composition or SDS concentration did not subjectively affect the quality of the cleared tissue. We did however find that an increase in SDS concentration (from 4% to 8%) slightly increased the speed of tissue clearing, and that hydrogel composition affected clearing times more substantially. The original hydrogel composition (A4B5P4) allowed 2 mm slices to clear in 5 weeks and whole adult mouse brains to clear in approximately 15 weeks. However, samples cleared much faster when prepared with hydrogel containing lower PFA and acrylamide concentrations (Table 1). The definition of “clear” is experiment specific because the amount of acceptable scatter depends on the brightness of the fluorescent probe, the resolution needed, and the microscope modality. For this reason, the results in Table 1 were not repeated, and are intended as a guide. All hydrogel compositions allowed for successful staining, and so subjectively, hydrogel composition does not appear to affect antigen preservation. However, tissue rigidity is affected, and care must be taken not to damage samples prepared with low-acrylamide hydrogel.

**Table 1:** Time taken to clear mouse brain hemispheres prepared with different hydrogel concentrations.

Hydrogel composition	Acrylamide [%]	Bis-acrylamide [%]	Paraformaldehyde [%]	Time taken for clearance [weeks]
A4B0P0	4	0.00	0	3
A1B5P1	1	0.05	1	5
A2B5P2	2	0.05	2	6
A4B5P4	4	0.05	4	12

### Tissue staining

#### Antibody staining

**Figure 2:**
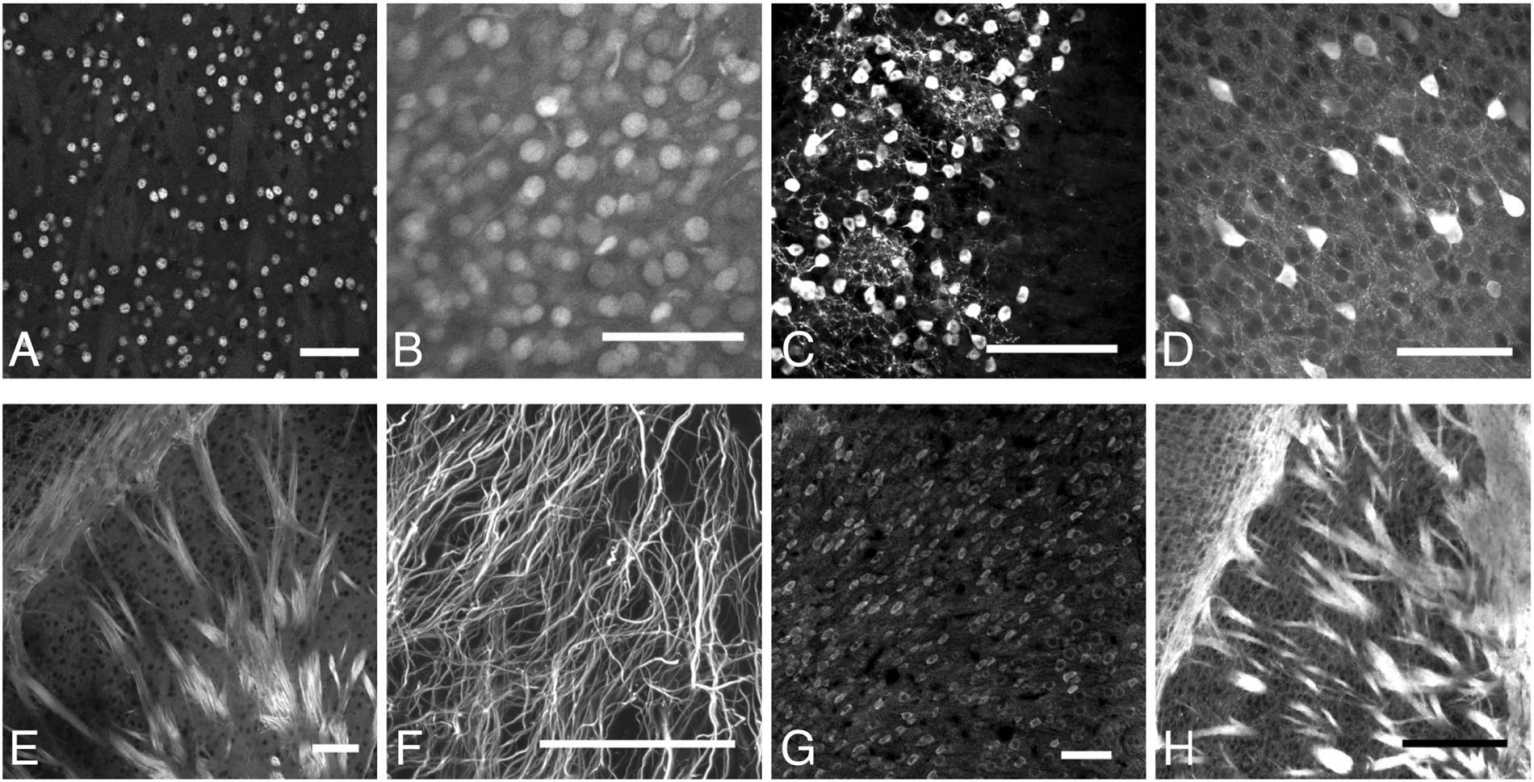
Examples of successful antibody stains in mouse brain tissue. Scale bars 100 μm. (**A)** CTIP2 staining of striatal spiny projection neurons, (**B)** CUX1 staining of layer II–IV cortical projection neurons, (**C)** Calbindin staining in olfactory bulb glomerular layer, (**D)** Parvalbumin staining of primary somatosensory cortical interneurons, (**E)** Neurofilament staining of striatal white matter tracts, (**F)** GFAP staining of cerebellar Bergmann glia, (**G)** NeuN staining in somatosensory cortex, (**H)** MBP staining of striatal white matter tracts.

Following tissue clearing, a number of different antibodies (Fig. 2, Supplementary Table 2) were tested, related to a variety of aspects of brain structure. Markers included cortical layer markers, striatal cell markers, inhibitory interneurons, synaptic markers, white matter markers and general cell type markers. Of these antibodies, eight produced reliable staining. These included the neuronal cell type markers CTIP2, CUX1, calbindin and parvalbumin along with the stains for neuronal projections (MBP and neurofilament) and general neuronal (NeuN) and astrocyte (GFAP) markers.

All appropriate secondary antibodies (Supplementary Table 3) tested worked well, and so it is thought that only the primary antibody choice is critical in cleared tissue.

#### Antibody penetration

**Figure 3:**
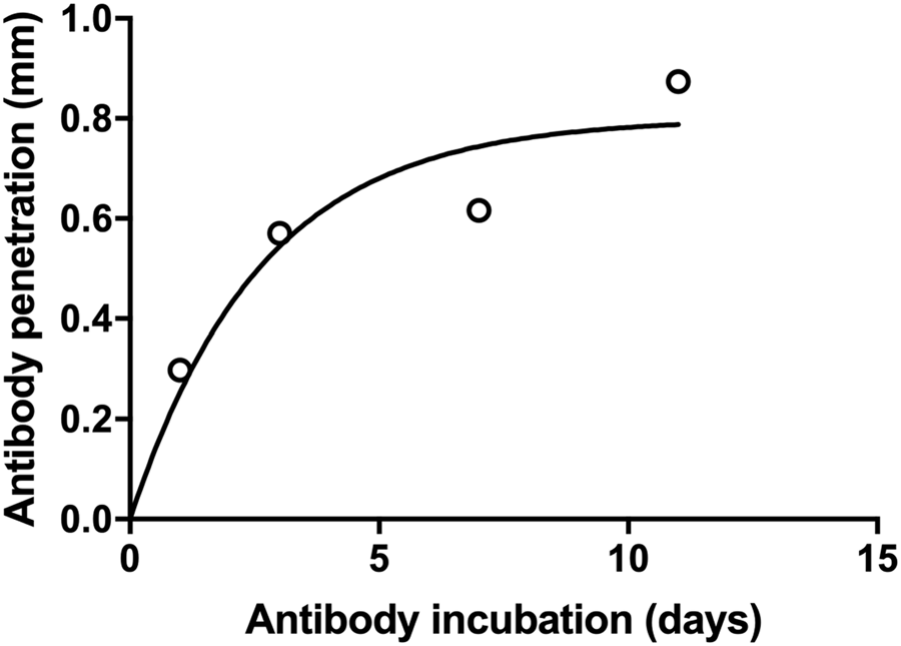
Antibody penetration depth as a function of incubation time (equal for primary and secondary antibodies), fitted with an exponential $(0.8-0.8{e}^{-0.38x})$.

To assess how much time antibodies took to diffuse into cleared tissue, the diffusion of the calbindin (rabbit) antibody was tested in mouse cortex (hydrogel composition — A4B5P4). This antibody was chosen as the protein is expressed relatively evenly across the cortex without being too dense (unlike neurofilament for example). Dense protein expression would complicate analysis, as antibody depletion would become the main factor limiting staining depth, rather than diffusion speed. To determine the speed of staining, the antibody incubation time (for primary and secondary antibody) was varied, and the staining depth (the distance into the tissue at which brightly positive cells could be seen) was measured. To ensure that differences in the level of tissue clearance did not affect antibody penetration, this was repeated in different sections of the same mouse brain, all the same thickness (1.5 mm), and cleared for the same length of time (3 weeks). Samples were incubated in antibody solutions for 1, 3, 7 or 11 days, and antibody penetration was measured. This was measured in four different cerebral cortical areas spread across each tissue section. Measurements were taken in deep cortical layers (to prevent any contribution of antibody diffusing from the cortical surface). The mean was taken, and this is plotted in Fig. 3.

#### Small molecule stains

**Figure 4:**
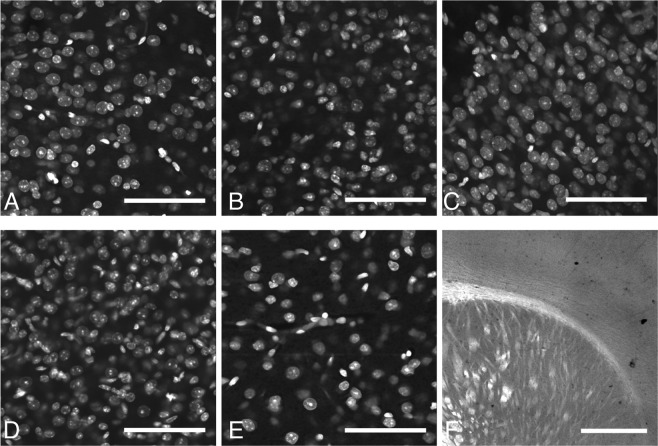
Other fluorescent stains in mouse brain tissue. All in primary somatosensory cortex and scale bars 100 μm other than fluromyelin (cortex and striatum scale bar 1 mm). (**A)** DAPI, (**B)** Propidium iodide, (**C**) SYTOX green, (**D)** SYTOX red, (**E)** Neurotrace red, (**F)** Fluromyelin green.

Due to the slow diffusion of antibodies into cleared brain tissue, low molecular weight, non-antibody based fluorescent stains were investigated. These stains could all be used to successfully stain an entire, intact mouse brain within 24 hours (e.g. Supplementary Fig. 1), and as such greatly increase the flexibility of hydrogel-based tissue clearing. These dyes included nucleic acid, Nissl and myelin stains (Fig. 4, Supplementary Table 4).

### Comparison to conventional fixation

One potential issue with hydrogel-based tissue clearing techniques is the expansion of the tissue that has been observed^31^. Although referenced in the literature, this tissue expansion has not been investigated in detail. All tissue processing techniques will affect brain structure in some way, and so tissue expansion is not necessarily a problem, as long as this tissue expansion is uniform across different brain areas and different spatial scales.

To investigate the effects of tissue clearing, cleared tissue was compared to that fixed only using PFA and not cleared. Two adult, female littermate mice were perfused with phosphate buffered saline (PBS) and 4% PFA. One brain was post-fixed in hydrogel, before both brains were cut into 500 μm coronal sections and the hydrogel-fixed tissue was cleared. Following clearing, the cleared tissue and uncleared were treated identically and stained for a selection of markers. It is not possible to measure every possible brain structural parameter in every brain area, and so initially two anatomical markers in two brain regions were investigated. The cerebral cortex and striatum were investigated because of their very different structure, and how this could affect structure following clearing. The striatum contains large numbers of myelinated fibre tracts (Fig. 2H) which have a very high lipid content. The lipid removal process (which is key to hydrogel-based tissue clearing) could therefore have a differential effect upon the striatum compared to the cortex which has a lower lipid content. The markers chosen were the antibody stains CTIP2 and parvalbumin. These were chosen because they are expressed densely in both areas, mark different cell types and because they allow the measurement of two key parts of tissue microstructure which may be affected differentially – nuclear volume (CTIP2) and whole-cell volume (parvalbumin).

**Figure 5:**
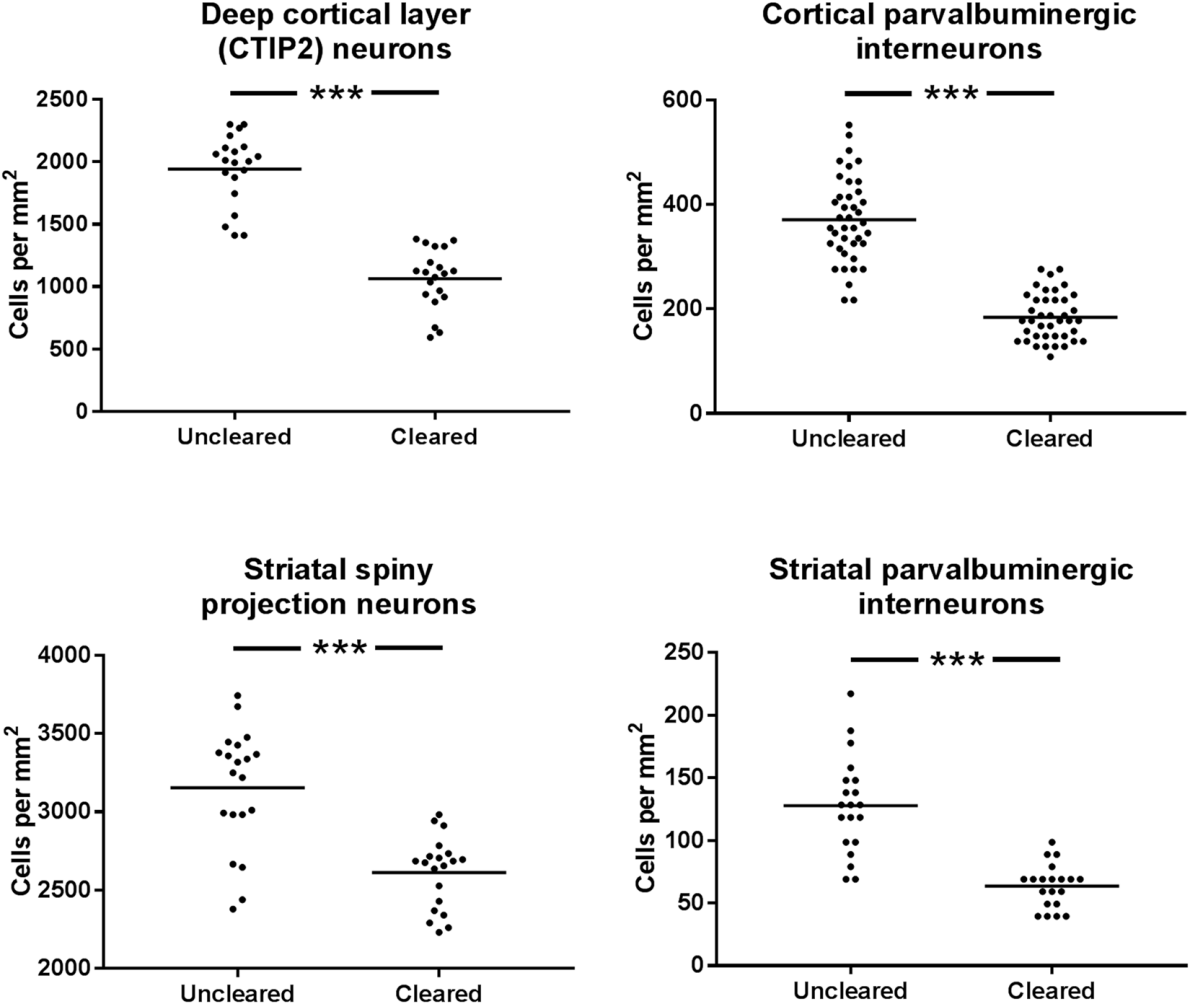
Cell counts of CTIP2- and parvalbumin-positive cells in cortex and striatum, comparison between uncleared and cleared mouse brain tissue. Mean shown as a horizontal line.

Two-dimensional (2D) images were acquired across the cortex (primary motor cortex and barrel cortex) and striatum to compare the cell density of CTIP2- and parvalbumin-positive cells. Two cleared and two uncleared slices (each pair from a single brain) were imaged for each cell type in each brain area, with 80 images taken of cortical parvalbumin-positive cells (40 cleared and 40 uncleared), and 40 images taken of each cortical CTIP2-positive cells, striatal CTIP2- and striatal parvalbumin-positive cells. In all cases, the cell density was lower in cleared than uncleared tissue (*p* < 0.001, Fig. 5), suggesting tissue expansion. Although it is possible that the same results could occur owing to reduced antibody staining efficiency, this was thought to be unlikely, as the staining intensity of the positive cells was comparable between groups.

**Figure 6:**
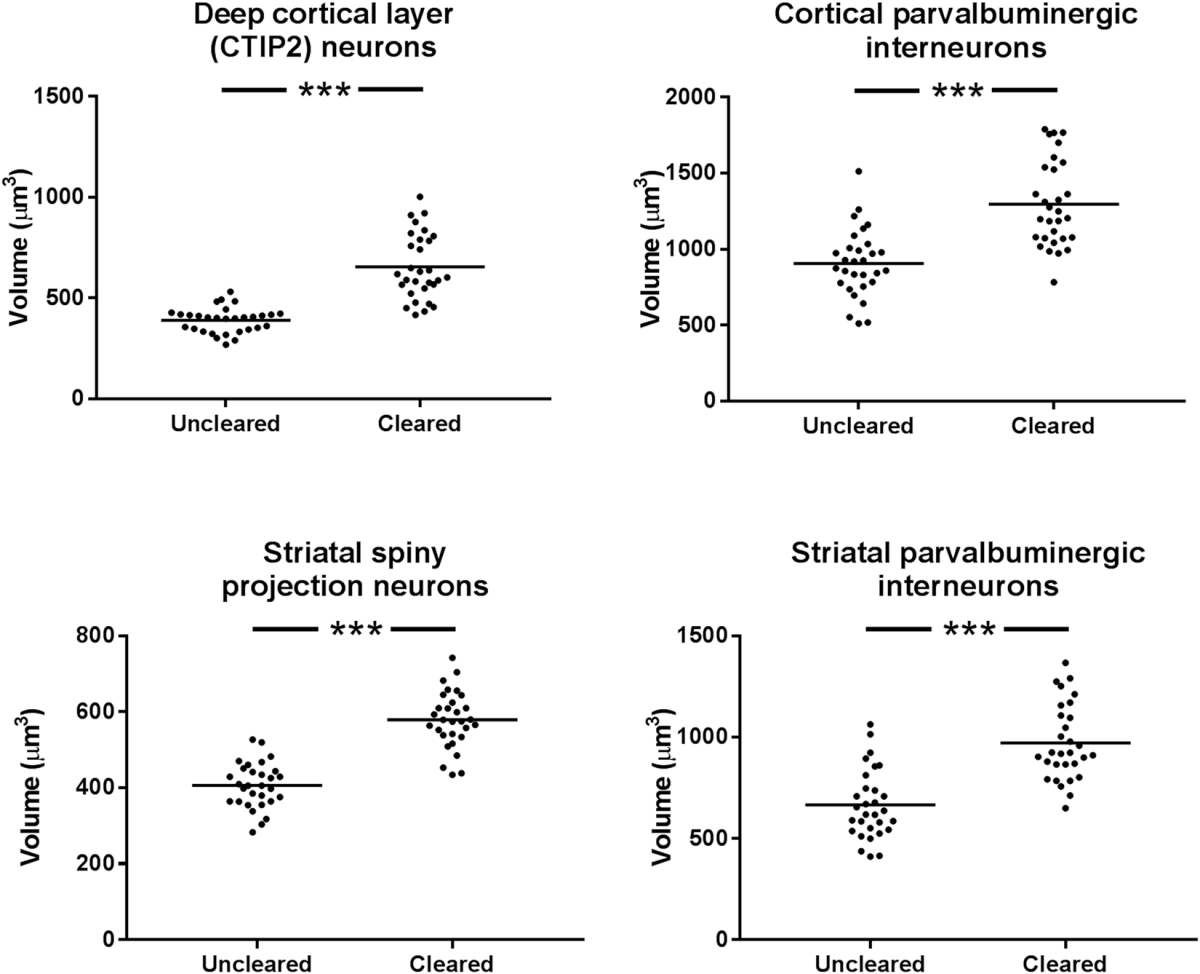
Volumes of CTIP2- and parvalbumin-positive cells in cortex and striatum, comparison between uncleared and cleared mouse brain tissue. Mean shown as a horizontal line.

To better understand whether these results were simply due to tissue expansion, and to assess whether this expansion occurred at different spatial scales, the volume of CTIP2-positive nuclei and parvalbumin-positive cells was investigated. The cell density result could have occurred due to uniform tissue expansion, or just expansion of the extracellular space. As before, two cleared sections and two uncleared sections were imaged for each cell type and each brain area. 30 cells in cleared tissue and 30 in uncleared tissue were imaged in three-dimensions (3D) for each cell type in each brain area, and the volumes (following manual segmentation) were compared between cleared and uncleared cells. In all cases, the cell volume was higher in cleared than in uncleared tissue (*p* < 0.001, Fig. 6).

**Figure 7:**
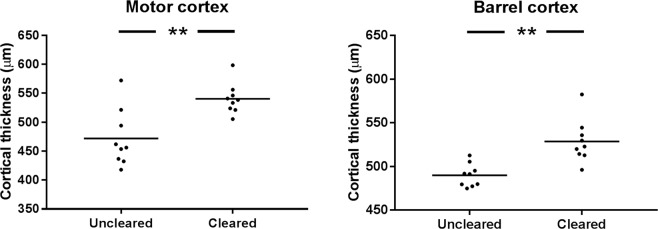
Comparison of cortical thickness in cleared and uncleared tissue, in motor and barrel cortices. Mean shown as a horizontal line.

An increase in cell volume alongside a decrease in cell density suggested that the findings were due to general tissue expansion that is uniform at different spatial scales. It was not possible to accurately measure the overall expansion of the brain due to small amounts of clearing-induced tissue deformation and tissue loss, and so DAPI staining was used to measure cortical thickness. Cortical thickness was measured in two areas (primary motor cortex and barrel cortex) in nine brain hemispheres in each group (cleared and uncleared). The cortices of cleared tissue were thicker in both motor cortex (*p* = 0.003) and barrel cortex (*p* = 0.001, Fig. 7), providing further evidence for general tissue expansion as the cause for the increased cell volume and reduced cell density.

Figures 5–7 appear to show that tissue expansion affects all the measures in a similar fashion. These techniques are used under the assumption that any effects they have upon tissue structure are uniform across spatial scales and that no brain area or cell type is differentially affected. To assess whether this was the case, the variation in the effect of clearing upon the different parameters discussed above was calculated. Firstly, the relative change in the mean of each parameter was calculated, to give a measure of the expansion (Table 2). For volume or thickness measurements this was calculated as the cleared value divided by the uncleared value, and the inverse for the density measurements. This measure was then normalised to expansion in one dimension, so the 2D density measures were square-rooted, and the 3D volume measurements were cube-rooted. This normalised expansion metric is termed the “adjusted expansion ratio”.

**Table 2:** Relative and adjusted expansion ratios for each individual measure comparing uncleared and cleared tissue.

	Measurement	Relative expansion	Adjusted expansion ratio
Cell density	Cortical CTIP2	1.83	1.35
	Cortical Parvalbumin	2.02	1.42
	Striatal CTIP2	1.21	1.10
	Striatal Parvalbumin	2.00	1.41
Cell volume	Cortical CTIP2	1.68	1.19
	Cortical Parvalbumin	1.43	1.13
	Striatal CTIP2	1.42	1.12
	Striatal Parvalbumin	1.46	1.13
Cortical thickness	Motor cortex	1.14	1.14
	Barrel cortex	1.08	1.08

This adjusted expansion ratio seems relatively similar (between 1.08 and 1.42) for all measures, but this does not give us any quantitative measure. To begin to understand whether the adjusted ratios are similar, and therefore whether the tissue expansion was uniform across these measures, the coefficient of variance (CV, ratio of the standard deviation to the mean) of these measures was then calculated from the adjusted expansion ratio. This measure, which we term the “clearing CV” is a single value which summarises how similar all of the adjusted expansion ratios are. If it is low, then clearing affected all the measures similarly (and tissue expansion can be thought of as uniform). If it is high, then the measures are affected very differently, and the tissue clearing must affect these measures in different ways.

Unfortunately, the nature of CVs is that there is no “rule of thumb” about what variance is high, low, acceptable or unacceptable. Therefore we sought to develop a cut off, a CV threshold that would tell us whether our clearing CV was low enough to be acceptable. In our experiment, we defined acceptable levels of variance to be that of each of the individual measures in uncleared tissue. DAPI and antibody staining in conventionally-prepared tissue is thought to be reliable enough that it does not introduce large amounts of variance. These techniques are used routinely in the field, and any variance they introduce is not required to be measured or explained when publishing work based on these methods. Measures of variance (in neuroscience) are usually carried out to understand the differences between experimental subjects, and the variance introduced by standard techniques such as immunohistochemistry is assumed to be low enough that it does not affect the outcomes of the study. We therefore propose that the variance of the measures in Figs 5–7 in conventionally prepared tissue (i.e. uncleared) in a single animal (removing any subject to subject variance) would give us an estimate (for this work at least) of what is “acceptable” variance. If the clearing CV (the variance introduced by clearing) is of a similar magnitude to the mean CV of each of these “standard” anatomical measures, then the variance introduced by clearing can be thought of as acting uniformly in different brain areas, and at different spatial scales.

**Figure 8:**
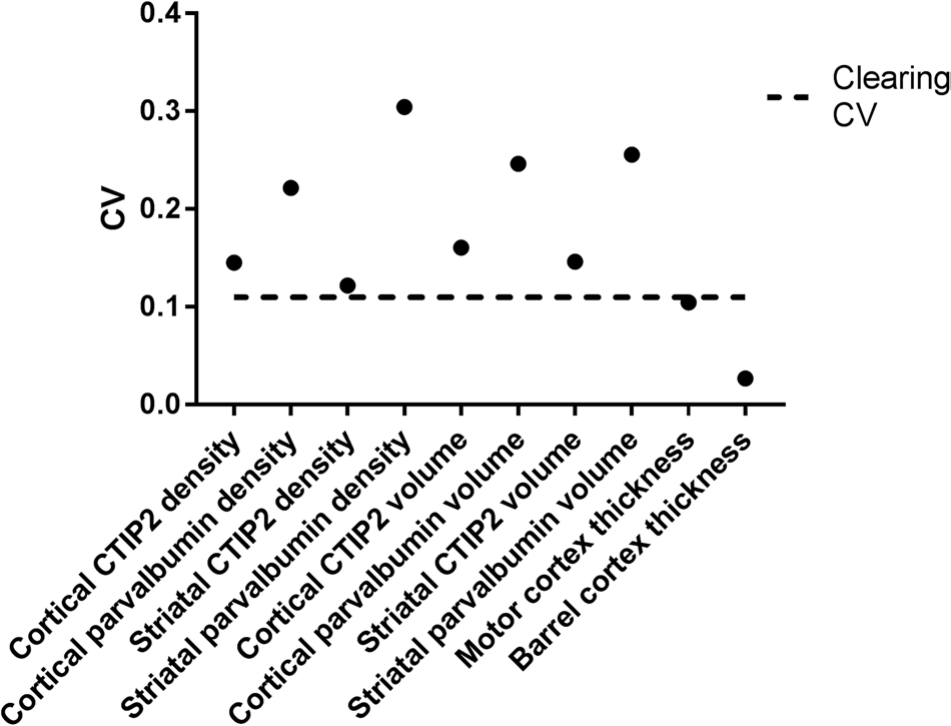
CVs for each measure in uncleared tissue with the CV due to clearing overlaid.

The clearing CV for tissue expansion was 0.110, which was lower than the CV for all the measures in uncleared tissue other than cortical thickness (0.104 & 0.027), as shown in Fig. 8. The mean CV for all of the measures in uncleared tissue was 0.173, considerably higher than the clearing CV. This approach is novel, and does not prove that clearing affects each metric uniformly, but it suggests that the clearing CV is similar to generally accepted variance in the field. Showing that the variance introduced by clearing is lower than variance which is routinely accepted in the field, goes some way to showing that the tissue expansion introduced by clearing is relatively uniform, and may eventually not need to be considered (as long as the proper controls are performed).

## Discussion

Hydrogel-based tissue clearing is a very reliable method, but one issue is compatibility with antibody staining. We found that out of 22 antibodies tested, only eight were successful. However, these eight were very reliable and are likely to be of great use in the neuroscience community. The clearing process is a very harsh one which may cause antigen damage and prevent antibody staining, but this does not explain why some antibodies worked and not others. There is no obvious common feature of the successful antibodies, and this remains something to be studied further.

Another issue with antibody staining is the time taken for uniform staining with high signal-to-noise (SNR). Conventional primary and secondary antibody technology is not suitable for diffusion through large volumes of tissue. There are many ways to potentially speed this up, but possibly at the expense of SNR or tissue damage. These methods include using fluorophore-conjugated primary antibodies, low molecular weight single-domain antibodies^32^ or the use of electric fields to accelerate diffusion^33,34^. Currently, none of these solutions are commercially available for most antibodies, and so researchers must rely on long incubation times of antibodies which practically limits the thickness of tissue that can be imaged.

The success of every small-molecule dye tested is potentially very useful. Cell nuclei stains have been used in conjunction with tissue clearing, but the Nissl and myelin stains have not yet (to our knowledge) been applied outside of method development. These stains can be used to stain much larger volumes of tissue than antibodies, and are much cheaper and easier to use. They are less specific than antibody stains, but in large tissue volumes they represent a great increase in specificity and resolution compared to competing techniques (e.g. magnetic resonance imaging).

We compared the variance of clearing-induced tissue expansion to acceptable levels of variance in the field. This approach was used due to a lack of standard statistical tests for this kind of work. While this does not prove that the tissue expansion is uniform, it does provide some sense of how uniform it is, and whether the variance introduced is low enough to not affect any biological conclusions. The variability of tissue expansion upon different measures in different brain areas is generally less than the natural variability associated with these measures in uncleared tissue. For this reason, it is not thought that tissue clearing will introduce any biases that could affect the interpretation of any results. It is also well known that many tissue fixatives (including PFA) can shrink brain tissue considerably^35^, so it may be that the swelling introduced by clearing compared to PFA-only fixation may return the tissue closer to the structure found *in vivo*.

Since the original description of hydrogel-based tissue clearing^17^, there have been close to a hundred publications applying and extending the method^36^, the majority of them in neuroscience. We have described a simple and reliable protocol allowing investigation of many common neuroanatomical features including cell densities, white matter and glial structure along with the morphology of a number of specific cell types. Very few of the existing papers have measured the effect upon morphology of these techniques, other than just describing tissue expansion very broadly. We have shown that although these methods do cause tissue expansion (at least compared to existing techniques), it can be thought of as happening relatively uniformly across spatial scales. Therefore as long as these methods are applied consistently, the results from them can be interpreted as reliably as with other, more established methods.

## Methods

All procedures were performed under local King’s College London Animal Welfare and Ethical Review Body approval and under UK Home Office project and personal licenses, where necessary, in accordance with the Animals (Scientific Procedures) Act 1986. All chemicals used are listed in Supplementary Table 1.

### Tissue clearing

C57BL/6J mice were euthanised by cervical dislocation, and brain tissue was rapidly dissected and incubated in ice-cold hydrogel solution. Hydrogel solutions were made up in PBS with 0.25% VA-044 photoinitiator (Wako Chemicals GmbH, DE) according to Table 1.

Samples were incubated in hydrogel solution (with gentle shaking) at 4 °C for one week. To prevent inhibition of the polymerisation by oxygen, the samples were degassed using nitrogen^17^. This process involves setting up a vacuum gas manifold connected to a vacuum desiccator. A tube containing the sample in hydrogel solution is held under strong vacuum for 10 minutes, before flushing the tube with oxygen-free nitrogen gas. This is repeated and then the tube is closed as quickly as possible to prevent any oxygen entering the tube.

The sample tube (with hydrogel solution) was then polymerised in a 37 °C water bath for 3 hours. It is recommended to use as large a water bath as possible, and to not use an incubator because the temperature and duration of polymerisation appear to have an effect upon the extent of polymerisation and eventual speed of tissue clearing and staining.

Once polymerisation is complete, the excess hydrogel will appear to have thickened or solidified, and the excess can be removed from the brain. The use of tissue paper is recommended to remove as much excess hydrogel as possible. If the consistency of the hydrogel has not changed, unless very low concentrations of PFA and acrylamide are used, it is likely that hydrogel polymerisation has failed. The most likely issue is the pH of the PFA solution if it is made up in the laboratory. A neutral pH is required for polymerisation (7.4 is used successfully in our laboratory).

Once excess hydrogel was removed, the brain was incubated in clearing solution (SDS in in 0.2 M boric acid, pH = 8.5) whole, as hemispheres, or in 500 μm (using a vibratome, VT1000S, Leica Biosystems GmbH, Germany), 1.5 mm or 2 mm (using a brain-slicing matrix^30^) sections. Clearing buffer was exchanged after 24 and 48 hours, and then weekly until samples became transparent. It is notably difficult to decide when to stop the clearing process, and tissue clarity eventually appears to plateau, leading to no changes from one week to the next. For this reason, the following rules were used:

- After the first 48 hours, only exchange clearing buffers (and therefore check clarity) every 7 days.
- Check tissue clarity in the same conditions such as in Fig. 1. Placing the samples in the same solution (e.g. clearing buffer) and on the same material (e.g. a backlit grid) greatly aids consistency in checking clarity.
- Stop clearing once the tissue does not obscure printed structures underneath.
- Compare the sample to other samples in the same study that have already been cleared for consistency.

In our laboratory, following these rules usually leads to all samples of the same type being cleared for the same length of time, and consistent staining and imaging quality. As long as the tissue is cleared to the point at which patterns can be seen through it without distortion (e.g. Fig. 1B) we do not observe any issues downstream with antibody staining.

After clearing, samples are washed in PBSTN_3_ (0.1% Tx100 and 0.01% sodium azide in PBS) for 48 hours (exchanging the solution twice per day) and stored until staining.

### Tissue staining

Unless otherwise specified, primary antibody staining was carried out at the concentrations listed in Supplementary Table 2, made up in PBSTN_3_ for 7 days. The volume of antibody solution was also chosen to be at least five times larger than the volume of tissue (e.g. 1 ml for a 2 mm coronal mouse brain section). To speed up antibody penetration and ensure even staining, antibody incubation was performed with gentle shaking at 37 °C. Secondary antibody staining was performed in the exact same conditions, for the same length of time with the appropriate AlexaFluor 488 conjugated secondary antibody (Life Technologies Ltd, UK) but at a concentration of 1:50. The use of a blocking agent (e.g. bovine serum albumin) has not shown any effect upon staining quality in cleared brain tissue, and so was not used.

Small molecule stains were also carried out in PBSTN_3_, but at room temperature (approximately 20 °C), and for 24 hours. All samples were thoroughly washed in PBSTN_3_ and then incubated in 85% glycerol in PBS for 24 hours prior to imaging.

### Imaging and data analysis

Antibody and dye optimisation imaging was carried out on a variety of confocal and multiphoton microscopes. The comparison between cleared and uncleared tissue was carried out on a Nikon Eclipse 80i C1 laser-scanning confocal microscope (Nikon Instruments Europe BV, NL) using the parameters in Supplementary Table 5. All images in this section were 512 × 512 pixels and taken with a 150 μm pinhole.

All images were analysed using ImageJ^37–40^. Cell densities were determined manually and cortical thickness measurements were taken by measuring the length of straight lines drawn across the cortex. To measure cell volumes, cells were segmented manually using the segmentation editor plugin^41^ and the volumes of the ROIs were calculated using the 3D ImageJ suite^42^. All image figures were generated using ImageJ and all statistics and plots were generated using Prism (Graphpad Software Inc, USA). In all cases, datapoints represent regions of interest, and not individual animals. Distributions were assessed for normality using the Shapiro-Wilk test and all were normal other than cortical CTIP2 cell counts in uncleared tissue. No analysis of correlation was performed to compare cleared and uncleared tissue. For ease of interpretation, all comparisons were carried out using the parametric t-test. Due to the notable general reduction in variance for some variables in cleared tissue, Welch’s adaptation was used throughout (**p < 0.01, ***p < 0.001). Full test statistics are shown in Supplementary Table 6.

## Supplementary information

Supplementary Information

## Data Availability

All raw data generated in the production of this manuscript is available on request.
