# Supplementary material for: Optimisation and validation of hydrogel-based brain tissue clearing shows uniform expansion across anatomical regions and spatial scales
Source: Sci Rep. 2019 Aug 19;9:12084. doi: 10.1038/s41598-019-48460-2 (PMC6700094; doi:10.1038/s41598-019-48460-2)
Supplement: Supplementary file 1 — Supplementary Information [file 41598_2019_48460_MOESM1_ESM.pdf]

Optimisation and validation of hydrogel-based brain tissue clearing shows uniform expansion across anatomical regions and spatial scales

Adam L. Tyson[1,2,3*], **Ayesha Akhtar**[1], **and Laura C. Andreae**[1,2+]

[1]Centre for Developmental Neurobiology, Institute of Psychiatry, Psychology and Neuroscience, King's College London, London, UK.
[2]MRC Centre for Neurodevelopmental Disorders, King's College London, London, UK.
[3]Present address: The Sainsbury Wellcome Centre for Neural Circuits and Behaviour, University College London, London, UK.
[*]adam.tyson@ucl.ac.uk
[+]laura.andreae@kcl.ac.uk

SUPPLEMENTARY INFORMATION

Material	Source
2% Bis-acrylamide	Bio-Rad (U.K.)
4',6-diamidino-2-phenylindole (DAPI)	Life Technologies (U.K.)
40% Acrylamide	Bio-Rad (U.K.)
Boric acid	Sigma-Aldrich (U.K.)
Fluromyelin green	Life Technologies (U.K.)
Glycerol	Fisher Scientific (U.K.)
Neurotrace red	Life Technologies (U.K.)
Paraformaldehyde	Sigma-Aldrich (U.K.)
Phosphate buffered saline tablets	Fisher Scientific (U.K.)
Propidum iodide	Life Technologies (U.K.)
Sodium azide	Sigma-Aldrich (U.K.)
Sodium dodecyl sulphate	Sigma-Aldrich (U.K.)
Sodium hydroxide	Sigma-Aldrich (U.K.)
SYTOX green nucleic acid stain	Life Technologies (U.K.)
SYTOX red nucleic acid stain	Life Technologies (U.K.)
Triton-X 100	Sigma-Aldrich (U.K.)
2,2'-Azobis[2-(2-imidazolin-2-yl) propane] dihydrochloride (VA-044)	Wako Chemicals (Germany)

Supplementary Table 1. Chemicals and materials used.

Antibody	Species	Isotype	Manufacturer	Code	Dilutions tested
**Calbindin D-28k**	Rabbit		Swant	CB38	**1:100**, 1:150
Calretitin	Rabbit		Chemicon	AB149	1:300
Calretitin	Rabbit		Swant	7699/3H	1:100
**CTIP2**	Rat	IgG2a	Abcam	AB18465	**1:100**, 1:150
**CUX1**	Rabbit		Santa Cruz Biotechnology	SC-13024	**1:50**, 1:100
CUX1	Rabbit		Proteintech	11733-1-AP	1:50, 1:100
GABA A α1	Rabbit		Millipore	AB5592	1:150
**Glial fibrillary acid protein (GFAP)**	Mouse	IgG1	Millipore	MAB360	**1:100**
Myelin basic protein	Rat	IgG2a	Millipore	MAB386	1:300
**Myelin basic protein (MBP)**	Rat	IgG2a	Abcam	AB7349	**1:100**
**NeuN**	Mouse	IgG1	Millipore	MAB377	**1:100**
**Neurofilament**	Chicken		Aves	NF-H	**1:100**
Neuropeptide Y	Rabbit		Diasorin	22940	1:300
Parvalbumin	Goat		Swant	PVG213	1:100
**Parvalbumin**	Rabbit		Abcam	AB11427	**1:100**
Parvalbumin	Mouse	IgG1	Swant	235	1:100
Parvalbumin	Goat		Abcam	AB32895	1:100
Pax6	Mouse	IgG1	Developmental studies hybridoma bank		1:50, 1:100
Somatostatin	Rat	IgG2b	Millipore	MAB354	1:100
Tyrosine hydroxylase	Rabbit		Millipore	AB152	1:600
VGLUT1	Rabbit		Synaptic Systems	135 303	1:250

Supplementary Table 2. Primary antibodies tested, successful antibodies and dilutions in bold.

Excitation wavelength (nm)	Species raised in	Species raised against	Code
488	Donkey	Rat IgG (H+L)	A-21208
488	Goat	Chicken IgY (H+L)	A-11039
488	Donkey	Rabbit IgG (H+L)	A-21206
488	Goat	Mouse IgG (H+L)	A-11001
555	Goat	Rabbit IgG (H+L)	A-21428
555	Goat	Mouse IgG (H+L)	A-21422
568	Donkey	Rabbit IgG (H+L)	A-10042
568	Goat	Rat IgG (H+L)	A-11077
568	Goat	Rabbit IgG (H+L)	A-11011
647	Goat	Chicken IgG (H+L)	A-21449

Supplementary Table 3. AlexaFluor secondary antibodies successfully tested (Life Technologies, U.K.)

Stain	Target	Successful concentrations
DAPI	Nucleic acids (cell nuclei)	3.6 µM to 36 µM
Propidum iodide	Nucleic acids (cell nuclei)	3 µM
Sytox green	Nucleic acids (cell nuclei)	5 µM
Sytox red	Nucleic acids (cell nuclei)	5 µM
Neurotrace red	Nissl bodies (neurons)	1:100
Fluromyelin green	Myelin (white matter)	1:100

Supplementary Table 4. Fluorescent small molecule dyes tested.

Anatomy	Objective lens	Numerical aperture	Illumination wavelength [nm]	Pixel dwell [µs]	Step size [µm]
Cell density	CFI Fluor 40XW	0.80	488	10.08	
Cell volume	CFI Plan Fluor 60XW	1.00	488	5.12	1.5
Cortical thickness	CFI Plan Fluor 4X	0.13	488	10.08	

Supplementary Table 5. Tissue clearing comparison microscopy acquisition parameters.

Cell density (cells per mm^3)		Mean	SD	Test result
Cortical CTIP2	Uncleared	1944	282	$t(37.1)=10.6$, $p=<0.001$
	Cleared	1064	240	
Cortical parvalbumin	Uncleared	371.0	82.1	$t(60.6)=12.6$, $p=<0.001$
	Cleared	184.4	45.2	
Striatal CTIP2	Uncleared	3154	384	$t(30.8)=5.43$, $p=<0.001$
	Cleared	2613	226	
Striatal parvalbumin	Uncleared	127.9	38.9	$t(26.3)=6.74$, $p=<0.001$
	Cleared	63.68	17.4	
Cell volume (μm^3)				
Cortical CTIP2	Uncleared	390.6	62.6	$t(37.4)=8.32$, $p=<0.001$
	Cleared	655.0	163	
Cortical parvalbumin	Uncleared	905.8	223	$t(55.0)=5.93$, $p=<0.001$
	Cleared	1296	283	
Striatal CTIP2	Uncleared	406.0	59.3	$t(55.3)=9.97$, $p=<0.001$
	Cleared	579.1	74.3	
Striatal parvalbumin	Uncleared	665.5	170	$t(57.5)=6.64$, $p=<0.001$
	Cleared	971.8	167	
Cortical thickness (μm)				
Motor cortex	Uncleared	471.8	49.2	$t(12.2)=3.69$, $p=0.003$
	Cleared	540.4	26.3	
Barrell cortex	Uncleared	489.8	13.2	$t(12.2)=4.20$, $p=0.001$
	Cleared	528.8	24.5	

Supplementary Table 6. Descriptive statistics and *t*-test results of the comparison between uncleared and cleared tissue.

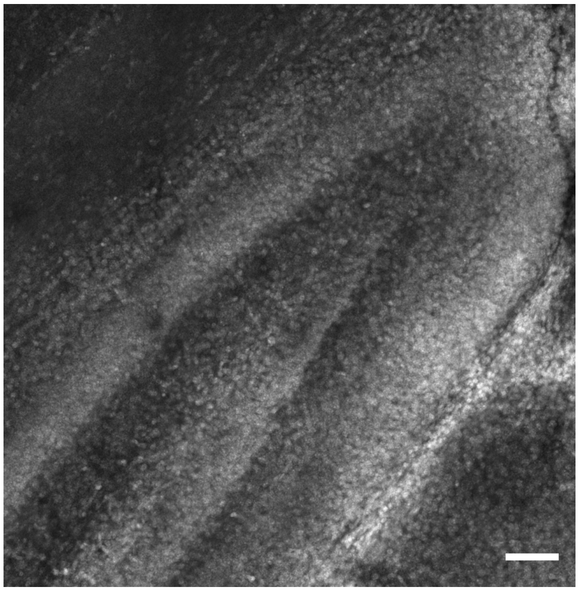

Supplementary Figure 1. DAPI staining in posterior hippocampus following 24 hour incubation of the intact, cleared brain. The brain was stained while intact, and then washed before sectioning for imaging.
